# Tissue-Specific Differences in Human Transfer RNA Expression

**DOI:** 10.1371/journal.pgen.0020221

**Published:** 2006-12-22

**Authors:** Kimberly A Dittmar, Jeffrey M Goodenbour, Tao Pan

**Affiliations:** 1 Department of Biochemistry and Molecular Biology, University of Chicago, Chicago, Illinois, United States of America; 2 Department of Human Genetics, University of Chicago, Chicago, Illinois, United States of America; North Carolina State University, United States of America

## Abstract

Over 450 transfer RNA (tRNA) genes have been annotated in the human genome. Reliable quantitation of tRNA levels in human samples using microarray methods presents a technical challenge. We have developed a microarray method to quantify tRNAs based on a fluorescent dye-labeling technique. The first-generation tRNA microarray consists of 42 probes for nuclear encoded tRNAs and 21 probes for mitochondrial encoded tRNAs. These probes cover tRNAs for all 20 amino acids and 11 isoacceptor families. Using this array, we report that the amounts of tRNA within the total cellular RNA vary widely among eight different human tissues. The brain expresses higher overall levels of nuclear encoded tRNAs than every tissue examined but one and higher levels of mitochondrial encoded tRNAs than every tissue examined. We found tissue-specific differences in the expression of individual tRNA species, and tRNAs decoding amino acids with similar chemical properties exhibited coordinated expression in distinct tissue types. Relative tRNA abundance exhibits a statistically significant correlation to the codon usage of a collection of highly expressed, tissue-specific genes in a subset of tissues or tRNA isoacceptors. Our findings demonstrate the existence of tissue-specific expression of tRNA species that strongly implicates a role for tRNA heterogeneity in regulating translation and possibly additional processes in vertebrate organisms.

## Synopsis

Transfer RNAs (tRNAs) translate the genetic code of genes into the amino acid sequence of proteins. Most amino acids have two or more codons. Every organism has multiple tRNA species reading the codons for the same amino acid (tRNA isoacceptors). In bacteria and yeast, differences in the relative abundance of tRNA isoacceptors have been found to affect the level of highly expressed proteins. This tRNA abundance–codon distribution relationship can have predictive power on the expression of genes based on their codon usages. Approximately 450 tRNA genes consisting of 49 isoacceptors and 274 different sequences have been annotated in the human genome. This work describes the first comparative analysis of tRNA expression levels in eight human tissues using microarray methods. The authors find significant, tissue-specific differences in the expression of tRNA species and coordinated expression among tRNAs decoding amino acids with similar chemical properties in distinct tissue types. Correlation of relative tRNA abundance versus the codon usage of highly expressed, tissue-specific genes can be found among a subset of tissues or tRNA isoacceptors. Differential tRNA expression in human tissues suggests that tRNA may play a unique role in regulating translation and possibly other processes in humans.

## Introduction

Human tissues contain common and distinct macromolecular components in varying amounts. Large-scale, high-throughput analyses of mRNA expression in human tissues show tissue-specific gene expression (e.g., [1–3]). Transfer RNA (tRNA) plays a central role in translating the mRNA sequence into the protein sequence. Approximately 450 tRNA genes have been annotated in the human genome [4,5] (http://lowelab.ucsc.edu/GtRNAdb/Hsapi). These tRNA genes are scattered throughout the genome and are present on all but the Y chromosome. Twenty-two additional tRNA genes are present in human mitochondrial DNA [6,7]. Thus, there are approximately 473 human tRNAs that are grouped into 49 isoacceptor families to decode the 21 amino acids specified by the genetic code (20 standard amino acids and selenocysteine).

To our knowledge, no systematic studies of tRNA expression among human tissues have been published. The dearth of information on tRNA expression is the result of technical and intellectual obstacles. Accurate quantitation of individual tRNA species is challenging due to the extensive secondary and tertiary structure of tRNA and numerous post-transcriptional modifications [8], both of which interfere with reverse transcription and hybridization of short oligonucleotides. Enthusiasm for tackling this challenge was low because prior to the human genome sequencing project, human tRNAs were considered to be no more diverse than those in unicellular organisms [8,9]. Complete sequencing of the human genome revealed, however, that over 270 different tRNA sequences are present among approximately 450 tRNA genes. Since only 61 possible anticodons are specified by the triplet code, there are many distinct tRNA species with identical anticodons, but they contain sequence differences in the tRNA body. This sequence diversity is not an inevitable result of genetic drift in multicopy genes over the course of evolution but may also be of some functional relevance [10]. In stark contrast to vertebrate genomes, there are only 51 different tRNA sequences among 274 yeast tRNA genes [4,5,11].

Why should the human genome contain such a diverse array of tRNA sequences? A compelling explanation is that controlling expression of individual tRNA species enables another level of translational control for specific gene products. In bacteria and yeast, differences in the relative abundance of tRNA isoacceptors for a given amino acid clearly impact the synthesis of highly expressed proteins [12,13]. Codon bias in tissue-specifically expressed genes have been reported, prompting the insight that such biases may be related to potential tissue-dependent differences in tRNA expression [14]. tRNA is also the dominant ligand for the elongation factor 1α (EF-1α). Given the myriad supratranslational functions of EF-1α [15,16] in cellular physiology, variations in tRNA expression could influence these processes which include bundling of actins and disassembly of microtubules [17,18]. For these reasons, even a rudimentary analysis of tRNA expression in human tissues could reveal novel aspects of human tRNA biology.

Here we describe the comparative analysis of tRNA levels in eight human tissues and two human cell lines using a microarray method adapted from our previously developed arrays for bacterial tRNAs [19,20]. Our human tRNA microarray contains 42 probes for nuclear encoded tRNAs and 21 probes for mitochondrial encoded tRNAs. These probes cover tRNAs for all amino acids with enough sequence differences to be uniquely distinguished. Our results show that tRNA levels vary widely among human tissues and coordinate according to the properties of their cognate amino acids. The differences in relative expression of tRNA isoacceptors in several tissues show statistically significant correlation to codon usage of a group of approximately 15 to 40 of tissue-specific genes that are expressed at the highest levels among tissue-specific genes.

## Results/Discussion

### Design and Specificity of Microarrays for Human tRNA

tRNA was quantified by taking advantage of its universally conserved 3′CCA sequence to attach a fluorescently labeled probe to tRNA present in total RNA prepared from tissues or cell lines. tRNA labeled in this manner was hybridized to DNA probes arrayed on glass slides. The brain sample was included in all hybridizations to correct for the variations in fluorescence labeling and array manufacturing. We used probes that are 70 to 80 nucleotides long, covering the length of the entire tRNA minus the conserved 3′CCA sequence. Probes at these lengths significantly increase hybridization efficiency and eliminate the sensitivity to potential variations in post-transcriptional modifications [19]. We designed the probes for nuclear tRNA genes to distinguish expression between tRNA isoacceptors, i.e., tRNAs with different anticodon sequences that read the codons for the same amino acid. Often, an isoacceptor family is encoded by multiple highly homologous genes. For example, five tRNA^Arg^ isoacceptors read the six arginine codons.

Our work on bacterial tRNAs showed that two tRNAs having more than ten different residues can be generally distinguished on a microarray, whereas significant cross-hybridization occurs when the sequence difference between two tRNAs is less than eight [19]. Sequence alignment by Clustal X [21] shows that there are sufficient sequence differences between tRNA^Arg^ isoacceptors (more than ten among 70 to 75 residues) to enable design of three isoacceptor probes that separately cover tRNA genes with ACG (modified to ICG [22]), CCT, or TCT anticodons. Since sequence differences between tRNA^Arg^ with CCG and TCG anticodons are insufficient (fewer than eight in 70 to 75 residues) to allow the design of two distinct probes, a single probe is used for these tRNAs. Thirty-seven probes are designed in this way to cover the 49 human tRNA isoacceptors plus the initiator tRNA^Met^ (Tables S1 and S2). The gene sequences for tRNA^Lys^(TTT), tRNA^Leu^(TAA), tRNA^Thr^(TGT), and tRNA^Thr^(CGT) isoacceptors are distinct enough that separate probes can be designed for their individual tRNA genes. The mitochondrial encoded tRNA sequences are sufficiently different so that 21 probes are designed to cover all mitochondrial tRNA genes except tRNA^Glu^ (Tables S3 and S4).

**Figure 1:**
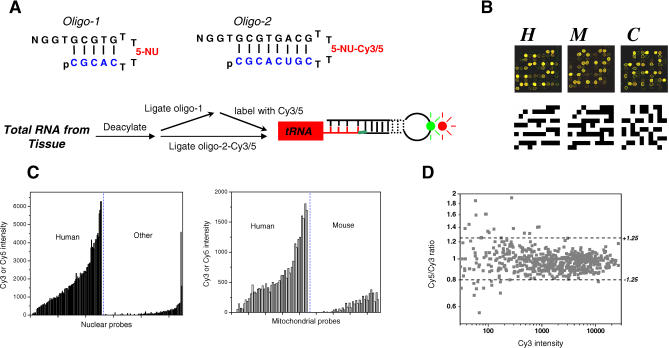
The tRNA Microarray (A) Schematics of fluorescence labeling of tRNA in a total RNA mixture. Ribonucleotides in the labeling oligos are shown in blue, and deoxyribonucleotides are shown in black. 5-NU, 5-allylamino-uridine; p, 5′ phosphate. All tissue samples are labeled with Cy3 and Cy5. At least two arrays are run for every sample pair. Array 1 used Cy3-labeled sample No. 1 and Cy5-labeled sample No. 2, and array 2 used Cy5-labeled sample No. 1 and Cy3-labeled sample No. 2. (B) Microarray images of one of the 32 blocks containing 100 spots hybridized with samples from HeLa (H), mouse kidney (M), and C. elegans whole animal (C). In the schematics below, black squares indicate the position of probes for the human (left), mouse (middle), and C. elegans (right) tRNAs. (C) Fluorescence intensity of a HeLa sample hybridized to the nuclear encoded tRNA probes (left) or mitochondrial encoded tRNA probes (right). “Human” indicates signals for the designated human probes; “other,” signals for *Drosophila* and C. elegans probes; and “mouse,” signals for mouse mitochondrial tRNA probes. (D) Dynamic range of Cy5/Cy3 ratio changes as a function of Cy3 intensity.

We established the specificity of the human tRNA array by examining the cross-hybridization of probes designed to detect tRNAs from different organisms (Figure 1). Based on sequence conservation, most of the 42 probes for human nuclear encoded tRNAs should hybridize to mouse tRNAs as well as to some *Drosophila* and Caenorhabditis elegans tRNAs [4,5]. Mitochondrial tRNA genes between human and mouse are sufficiently unique that 18 distinct probes for mouse mitochondrial tRNAs are present on the array. Approximately two-thirds of the *Drosophila* tRNAs and one-third of the C. elegans tRNAs have sufficient sequence similarity to human tRNAs that the same probes are used for these tRNAs. Ten separate probes for *Drosophila* and 34 probes for C. elegans tRNAs are also included on the array.

The microarray was tested using total RNA isolated from HeLa cells, mouse kidney, and entire *C. elegans.* Figure 1B shows one of the representative 32 blocks on the microarray. This block contains two to four repeats each of nine human probes, two mouse mitochondrial probes, six *Drosophila* or C. elegans probes, three probes as negative controls, and four blank spots. When HeLa total RNA was used, eight of nine human probes showed signals ranging from weak to strong. No signals were detected from the two mouse mitochondrial tRNA probes. Weak signals could be seen on one of the three negative control probes and two of six *Drosophila* and C. elegans probes. When mouse kidney total RNA was applied to the array, the two mouse mitochondrial tRNA probes showed intermediate to strong signals. When C. elegans total RNA was applied, all ten homologous probes showed weak to strong signals, with only a lone *Drosophila* probe showing a weak signal among the non–C. elegans probes. Using HeLa RNA, probes for human tRNAs show significantly higher hybridization signals than nonhuman probes (Figure 1C). Hybridization to all but one nonhuman probe yields signals that are lower than 10% of the strongest signals among the human tRNA probes. A single exception is the strong hybridization to the C. elegans tRNA^His^ probe which is not predicted by sequence similarity. These results show that the specificity of the microarray is sufficiently high given the extensive conservation among tRNAs between these organisms and supports its validity for measuring specific differences in tRNA expression.

To determine the appropriate dynamic range for the fluorescent dye ratios, serial dilutions of total RNA from HeLa were performed (Figure 1D). The dynamic range is at least two orders of magnitude as the dye ratios remain within a constant threshold of 1.25-fold. This result also suggests a detection limit of 1.25-fold for measured changes in tRNA abundance between two human samples. In bacteria and yeast, the relative abundance between tRNA members in an isoacceptor family ranges from 1-fold to 20-fold [23–26]. Our microarray is clearly capable of detecting differences within this range.

### tRNA Expression in Human Tissues and Cell Lines

In unicellular prokaryotes and eukaryotes, the abundance of tRNA isoacceptors is correlated with codon preferences among genes encoding highly expressed proteins, e.g., ribosomal proteins [11,27,28]. Mining mRNA microarray expression data, Plotkin et al. [14] reported the existence of tissue-based codon bias in paralogous genes; they proposed that this codon bias is related to tissue-specific differences in the abundance of corresponding decoding tRNAs. To explore this intriguing hypothesis, we used our microarray to measure the relative tRNA expression between eight tissues: brain, liver, vulva, testis and ovary, thymus, lymph node, and spleen. We included the latter three immune tissues based on an unexpected consequence of human genome sequencing: the single largest cluster of tRNA genes resides in the gene cluster of the major histocompatibility complex (also known as the human leukocyte antigen complex) [29]. The existence of one-third of all human tRNA genes in the human leukocyte antigen complex suggests that expression of these genes may be related to immune system function. For example, a high expression level of tRNA in this region may facilitate high expression of histocompatibility complex genes following a signaling event [29].

**Figure 2:**
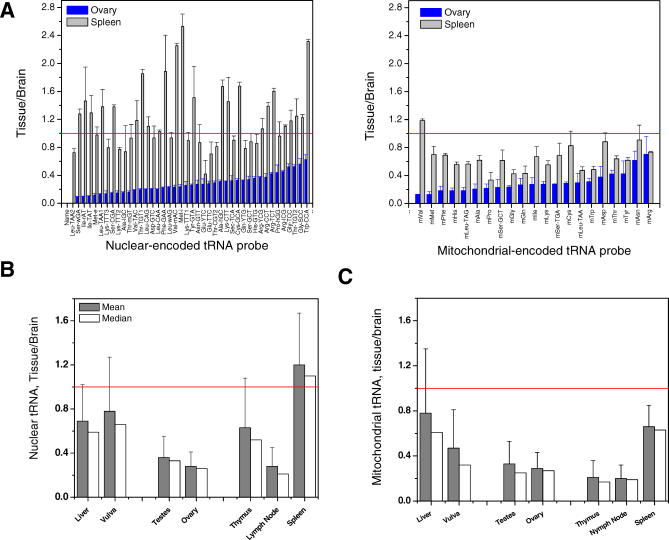
Overview of Relative tRNA Abundance among Eight Human Tissues Red line indicates the same level as brain. (A) Relative ratios of each human tRNA probe for ovary or spleen versus brain sorted according to the ratios for ovary. Left, nuclear encoded tRNAs; right, mitochondrial encoded tRNAs. (B) Mean and median values of the nuclear tRNA probes for seven tissues versus brain. (C) Mean and median values of the mitochondrial tRNA probes.

Microarray results show overall variations in the expression levels of tRNA among different tissues (Figures 2 and S1). For example, all tRNAs in ovary have lower levels relative to brain. Some tRNAs in spleen have higher, while others have lower levels, compared to those in brain (Figure 2A). Within individual tissues, the maximal differences between the relative tRNA levels can be as large as approximately tenfold (e.g., vulva, thymus) or only approximately threefold (e.g., testis).

Nuclear and mitochondrial encoded tRNA levels can be approximated separately by the mean and median tissue-to-brain ratio (Figure 2B and 2C). Liver and vulva have approximately two-thirds the amount of tRNA present in brain, while the reproductive tissues of testis and ovary express approximately one-third as much tRNA as the brain. Among the three immune tissues, thymus is comparable to liver and vulva, lymph node is similar to the reproductive tissues, while spleen has similar tRNA levels as the brain. On the other hand, the relative mitochondrial encoded tRNA levels in all seven tissues are lower than that in brain, a result that may reflect high mitochondrial translation activity in the brain (e.g., [30]).

Since mature tRNA in human is thought to be very stable, total tRNA levels likely reflect tRNA transcription rates [28]. tRNA is transcribed by multisubunit complexes of RNA polymerase III, TFIIIB and TFIIIC [31,32]. Varying the abundance of one or more of these protein factors as suggested by results from mRNA expression arrays [1,2] could lead to varying levels of transcription among tRNA genes. On the other hand, the levels or activities of these subunit components can also be controlled post-trascriptionally and post-translationally. Understanding the basis for tissue specific differences in total tRNA awaits direct experimental examination of all of the potential factors.

**Figure 3:**
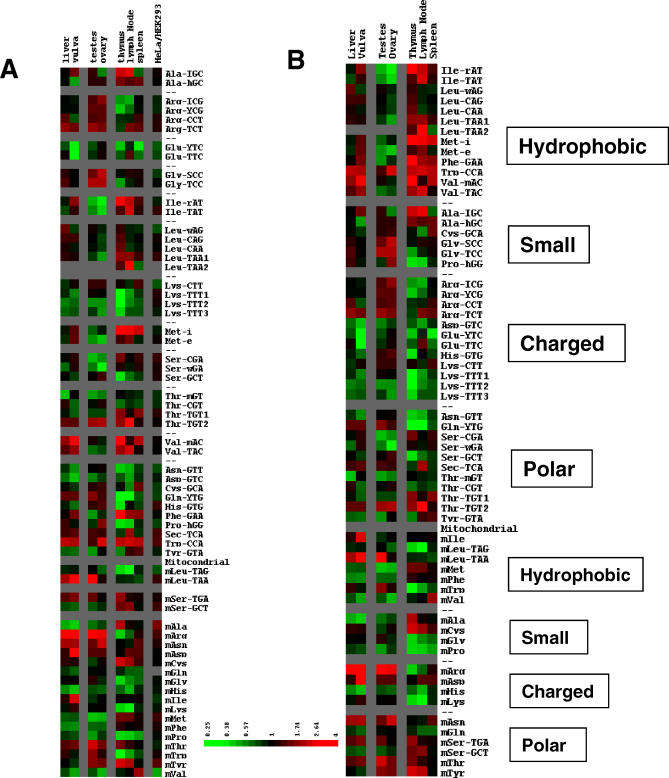
Comparative Expression of Nuclear and Mitochondrial Encoded tRNAs among Eight Human Tissues and Two Cell Lines Shown as TreeView Images [37] All tissue data are normalized to the median ratio in Figure 2B and 2C. The mean and median values for the HeLa/HEK293 cell lines are 1.19 ± 0.22 and 1.20 for nuclear tRNA probes and 1.48 ± 0.37 and 1.42 for mitochondrial tRNA probes. Green indicates decreased level of expression; red, increased level of expression in the indicated tissue over brain; gray, not determined due to very low signal intensity. Data are grouped according to codon-reading abilities (isoacceptors) (A) and corresponding amino acid types (B).

Large differences in the relative abundance of individual tRNAs from brain versus other tissues (e.g., ovary and spleen, Figure 2A) are observed. For example, both tRNA^Ile^ isoacceptors in ovary are expressed at only one-tenth of the level in brain, but these tRNAs in spleen are expressed above their levels in brain. To facilitate evaluation of tissue-specific differences in relative levels of individual tRNAs (Figure 3), we normalized fluorescent ratios internally to the separate median values among the nuclear- (Figure 2B) and the mitochondrial- (Figure 2C) encoded tRNAs.

Variations in the relative expression of tRNA isoacceptors among tissues are readily observed, suggesting a possible relationship between tRNA abundance and codon usage among different tissues (Figure 3 and Table 1). For example, among the members of the tRNA^Arg^ isoacceptor family, four probes separate the five isoacceptor groups according to their codon-reading capabilities: Arg-ICG reads CGU/C; Arg-YCG reads CGA/G; Arg-CCT reads only AGG; and Arg-TCT reads primarily AGA codons. A clear difference among these tRNA^Arg^ isoacceptors can be seen among the brain and the liver, thymus, and lymph node. Arg-TCT and Arg-CCT are preferred over Arg-ICG and Arg-YCG in these nonbrain tissues, suggesting a possible preference in reading AGA and AGG codons. Another example is the tRNA^Lys^ isoacceptor family. The AAG-reading isoacceptor is present in higher amounts than the AAA-reading isoacceptor in almost all tissues. This difference is particularly pronounced in vulva, thymus, and lymph node. The same type of analysis can be applied to tRNA isoacceptors for the glycine, isoleucine, leucine, serine, threonine, and valine families (Table 1).

**Table 1:**
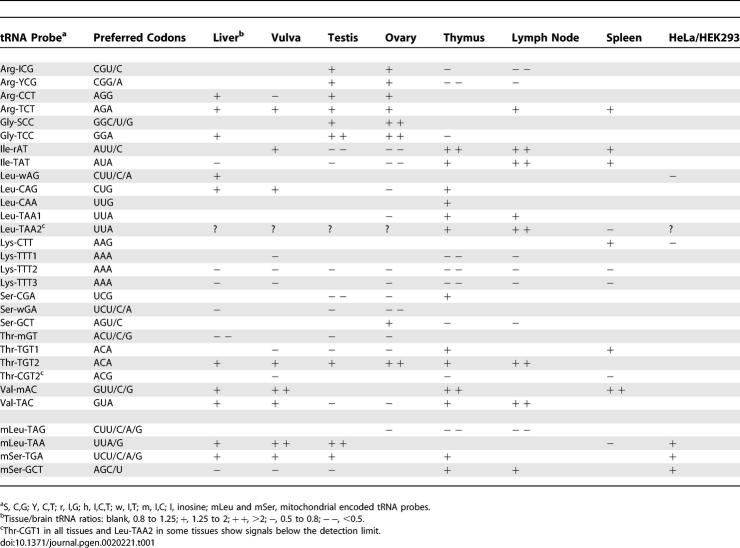
Suggested Codon Usage Preferences in Each Tissue Relative to Brain According to tRNA Expression

The relative expression of nuclear encoded tRNAs in two commonly used cell lines (HeLa and HEK293) differs by less than twofold and is quite similar among the isoacceptors (Figures 3 and S1 and Table 1). This result suggests that tRNA isoacceptor levels do not play a major role in differential protein expression in these cell lines, even though they are derived from different tissues (cervix and embryonic kidney). This may also be a result of nontissue processes such as the immortalized nature of these lines.

Interesting trends can be observed on the basis of the cognate amino acid properties of tRNA (Figure 3B). tRNAs and their corresponding amino acids can be divided into four groups: hydrophobic (Ile, Leu, Met, Phe, Trp, and Val), small (Ala, Cys, Gly, and Pro), charged (Arg, Asp, Glu, His, and Lys), and polar (Asn, Gln, Ser, Se-Cys, Thr, and Tyr). Among the nuclear encoded tRNAs, the three immune tissues (thymus, lymph node, and spleen) contain increased levels of tRNA for the hydrophobic group and decreased levels of tRNA for the charged group compared to brain. In contrast, the two reproductive tissues (testis and ovary) contain decreased levels of tRNA for the hydrophobic group and increased levels of tRNA for the small group. Trends for the liver and vulva are more similar to those of the immune tissues, with the increase in the tRNA for the hydrophobic group less pronounced. In general, brain contains increased levels of tRNA for the charged group (except for some tRNA^Arg^ isoacceptors) and for the polar group (except for some tRNA^Thr^ isoacceptors). The mitochondrial encoded tRNAs show distinctly different trends compared to the nuclear encoded tRNAs. The three immune tissues have decreased levels of mitochondrial tRNA for the hydrophobic group but comparable or increased levels of mitochondrial tRNA for the polar group relative to those in brain. The two reproductive tissues have decreased levels of mitochondrial tRNA for the small group than those in brain. These differences may reflect the amino acid availability in these tissues, although how this affects RNA polymerase III transcription is unclear.

### Correlating Relative tRNA Abundance to Codon Usage of Tissue-Specific Gene

The differential expression of tRNA isoacceptors can potentially be used to control translation via the codon usage of specific genes. However, it is unclear to which genes and at which stages of cellular development and differentiation this mechanism may be applied. Codon preferences are clearly present in some tissue-specifically expressed genes [14], although this bias has also been suggested to represent regional variations rather than selection for translational performance [33].

**Figure 4:**
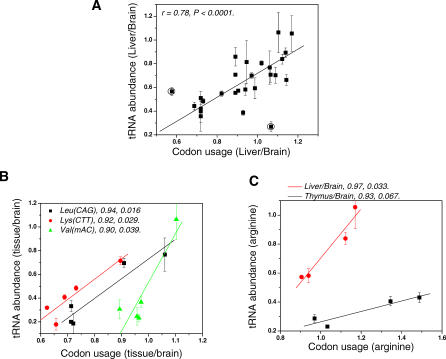
Linear Correlation of Relative tRNA Abundance to Codon Usage of Tissue-Specific Genes that Are Expressed at High Levels The *r*- and *p*-values are indicated in each graph. (A) Liver/brain, all data points. Excluding the two outlying data points (circled) gives a linear fit with *r* = 0.78, *p* < 0.0001, and a slope of 1.0 ± 0.2. Inclusion of these two data points gives a linear fit with *r* = 0.62, *p* = 0.00098, and a slope of 0.8 ± 0.2. (B) Three individual isoacceptors across all five tissues show linear correlation with *r* = 0.90 to 0.94 and *p* = 0.016 to 0.039. (C) Two sets of four tRNA^Arg^ isoacceptors in liver and thymus show linear correlation with *r* = 0.93 and 0.97 and *p* = 0.067 and 0.033, respectively.

We attempted to find potential correlations between the codon usage of tissue-specifically expressed genes to the relative tRNA abundance among these tissues (Figure 4). In bacteria and yeast, correlations of the abundance of tRNA isoacceptors to codon usage are derived primarily from genes that are translated at high levels (e.g., ribosomal proteins). Adapting this strategy, thresholds were algorithmically established to determine the top 13 to 43 tissue-specific transcripts according to the Human GeneAtlas GNF1H gcRMA dataset at http://symatlas.gnf.org/SymAtlas [2]. Gene sequences for the most highly expressed tissue-specific transcripts were then compiled and analyzed for codon content at http://bioinformatics.org/sms [34]. Some gene information was not included due to ambiguity of data or nomenclature. Table 2 lists the tissue-specific threshold expression levels used for this analysis and the tabulated gene and codon counts. The dataset contains 7,737 to 21,163 codons for six tissues: brain, liver, testis, ovary, thymus, and lymph node (we were unable to carry out this analysis for vulva and spleen). To mimic the relative tRNA abundance measurements, the raw codon count is normalized to the total number of codons in every tissue, and the sum of each normalized codon count from nonbrain tissues is divided by the sum of the same count from brain (Tables S5 and S6).

**Table 2:**
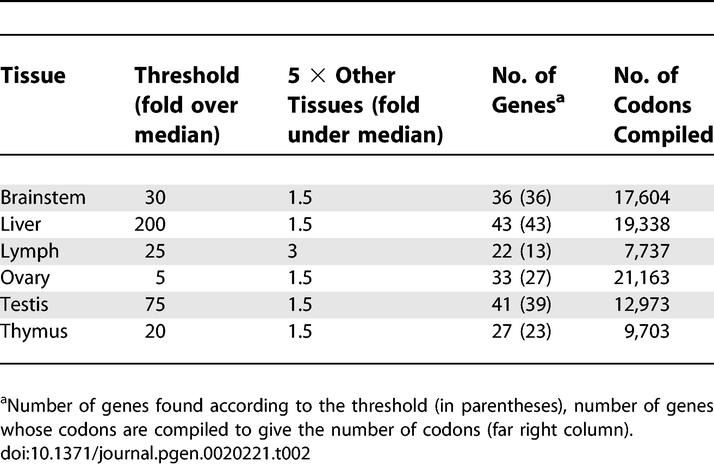
Expression Threshold Used in the Compilation of Codon Usages for Tissue-Specific Genes

The correlation of codon usage versus relative tRNA abundance (not divided by median values) was explored in three ways. First, all data points from the same tissue are plotted. For liver and brain, a linear fit of this plot gives an *r*-value of 0.78 and a *p*-value of <0.0001, a statistically significant correlation (Figure 4A). Similar correlations for testis, ovary, thymus, lymph node, and brain are much weaker and not statistically relevant (unpublished data). The fold over median expression threshold for liver is the highest among all tissues examined (Table 2), suggesting that the liver-specific genes are expressed at much higher levels than other tissue-specific genes. The high expression levels of liver-specific genes may explain why a significant tRNA abundance–codon usage correlation is only found in liver versus brain. Second, data points for each tRNA isoacceptor are plotted across five tissues. Linear correlations for three tRNA isoacceptors [tRNA^Leu^(CAG); tRNA^Lys^(CTT), and tRNA^Val^(mAC)] can be found with *r*-values from 0.90 to 0.94 and *p*-values from 0.016 to 0.039 (Figure 4B). Similar correlations for other isoacceptors are not statistically significant. Third, only two amino acids (arginine and leucine) have more than three data points and they are plotted for each tissue. Linear correlations among the four tRNA^Arg^ for two tissues (liver and thymus) can be found with *r*-values of 0.93 and 0.97 and *p*-values of 0.067 and 0.033, respectively (Figure 4C). Similar correlations for other tissues are much weaker.

### Conclusions

We have found that human tRNA expression varies by as much as tenfold among human tissues. Given the central role of tRNA in protein synthesis, this wide variation of tRNA abundance may reflect translational control via the availability of certain tRNAs. Since tRNA is the dominant ligand for the multitasking protein EF-1α, variations in tRNA levels may provide a mechanism to link translation with the dynamics of the cytoskeleton. Transcriptional control of tRNA genes may therefore play a role in the function of human tissues or possibly in cellular development and differentiation. tRNA abundance may also play a role in translational control of highly expressed, tissue-specific genes via their codon usage. Determination of tRNA abundance and charging levels [20] for differentiating cells or cells undergoing adaptation may reveal previously unseen connections between translation and other cellular processes.

## Materials and Methods

### Materials.

Total RNA from eight human tissues was purchased from Stratagene (http://www.stratagene.com): brain (No. 540005, No. 735006), liver (No. 735017), vulva (No. 735067), testis (No. 735064), ovary (No. 735260), thymus (No. 540141), lymph node (No. 540021), and spleen (No. 540035). After the microarray measurements were completed, it was found that the tissue RNA samples from Stratagene underwent an LiCl precipitation step which is known to result in the loss of small RNAs [35]. We subsequently found that the loss of tRNA is quite minimal when the concentration of the total RNA was greater than 1.5 μg/μl prior to the addition of LiCl and that the total amount of tRNA in brain was similar to that in HeLa isolated using a different protocol (Figure S2). The Stratagene RNAs we used are certified for microRNA studies. It is clear that under certain conditions, LiCl can precipitate microRNAs that are 3.5 times smaller than tRNAs. These results lend confidence that it is possible to perform LiCl precipitation and not affect tRNA studies.

The total RNA from the HeLa and HEK293 cell lines were obtained using RNAwiz (Ambion, http://www.ambion.com) according to manufacturer's manuals. This procedure does not include LiCl precipitation or other known steps biasing against tRNAs.

The three tRNA standards, E. coli tRNA^Lys^ (No. R6018), E. coli tRNA^Tyr^ (No. R0258), and yeast tRNA^Phe^ (No. R4018), were purchased from Sigma-Aldrich (http://www.sigmaaldrich.com) and used without further purifications.

### tRNA microarrays.

The microarray experiment consists of four steps starting from total RNA: (i) deacylation to remove remaining amino acids attached to the tRNA, (ii) selective Cy3/Cy5 labeling of tRNA, (iii) hybridization with prefabricated arrays, and (iv) data analysis.

For deacylation, 0.25 μg/μl total RNA premixed with the three tRNA standards at 0.17 μM each was incubated in 100 mM Tris-HCl (pH 9.0) at 37 °C for 30 min. The solution was neutralized by the addition of an equal volume of 100 mM Na-acetate/acetic acid (pH 4.8) plus 100 mM NaCl, followed by ethanol precipitation. Deacylated total RNA was dissolved in water, and its integrity was examined using agarose gel electrophoresis.

For Cy3/Cy5 labeling, tRNA in the total RNA mixture was selectively labeled with either Cy3 or Cy5 fluorophore using an enzymatic ligation method described previously [19,20]. The ligation reaction depends on the presence of the universally conserved 3′CCA nucleotides in every tRNA. Two different labeling oligonucleotides for the T4 DNA ligase were used in this work (Figure 1A). Both oligonucleotides are designed and shown to have insignificant bias for Cy3 and Cy5 dyes (Figure 1B and unpublished data). Oligo-1 contains 5-aminoallyl-U, and the Cy3 and Cy5 fluorophores were attached after the ligation step upon reactions with succimidyl esters of Cy3 or Cy5 (Amersham Biosciences, http://www.amersham.com, described in [20]). Oligo-2 contains an 8–base pair hybrid helix and either a Cy3 or Cy5 fluorophore preattached in the loop. The ligation reaction used an approximately 1 μM concentration of purified T4 DNA ligase for Oligo-1 but only 0.5 U/μl T4 DNA ligase (US Biochemicals, http://www.usbweb.com) for Oligo-2. Hence, the ligation of Oligo-2 required substantially less T4 DNA ligase compared to Oligo-1.

Hybridization was performed at 60 °C overnight with 1 μg each of Cy3- or Cy5-labeled total RNA mixture using Oligo-1 and 1 μg of Cy3-labeled total RNA and 2.5 μg of Cy5-labeled total RNA using Oligo-2 (because only 40% of Oligo-2 used in this work contained the Cy5 fluorophore). Multiple arrays were run using the brain reference sample labeled with either Cy3 or Cy5.

The microarray printing and hybridization conditions were the same as those in bacterial tRNA studies [19,20]. DNA oligonucleotide probes were designed on the basis of the 2001 version of the human genome [36]. Subsequent revision of the human genome sequences and tRNA annotations showed that 136 probes are useful for our study. They included 42 probes for human nuclear encoded tRNA genes, 21 probes for human mitochondrial encoded tRNA genes, 18 probes for mouse mitochondrial encoded tRNA genes, ten probes for *Drosophila* nuclear tRNA genes, 34 probes for C. elegans nuclear tRNA genes, three probes for bacterial and yeast tRNA standards, and eight probes for human tRNA hybridization controls. Nonhuman probes were used as specificity controls for hybridization of human samples. Eighteen replicates of each probe were printed on each array. The descriptions and sequences of the DNA oligonucleotide probes used for human nuclear and mitochondrial tRNA genes are provided in Tables S1 through S4.

For data analysis, arrays were scanned using GenePix 4000b scanner (Axon Instruments, http://www.axon.com) to obtain fluorescence intensities and the Cy5/Cy3 ratio per pixel at each probe spot. The averaged Cy5/Cy3 ratio per pixel at each probe spot was first normalized to an averaged value of the three tRNA standards prior to subsequent analysis. For all tissue samples, the brain total RNA was used as the reference sample at equal amounts of total RNA as determined by the UV absorbance. tRNA constitutes up to 15% of total RNA.

## Supporting Information

Figure S1 Relative Ratios of Each Human tRNA Probe for Liver, Vulva, Testis, Thymus, and Lymph Node versus Brain (55 KB PDF)

Figure S2 Recovery of tRNA Following LiCl Precipitation (36 KB PDF)

Table S1 Human Probes for Chromosomal Encoded tRNA Genes (10 KB PDF)

Table S2 Sequences of Human Probes for Chromosomal Encoded tRNA Genes (17 KB PDF)

Table S3 Human Probes for Mitochondrial Encoded tRNA Genes (8 KB PDF)

Table S4 Sequences of Human Probes for Mitochondrial Encoded tRNA Genes (12 KB PDF)

Table S5 Gene Sequences for Tissue-Specifically Expressed Gene According to mRNA Expression Data (429 KB XLS)

Table S6 Averaged Ratios of tRNA Abundance for Seven Tissues Relative to Brain (34 KB XLS)

## Abbreviations

EF-1α: elongation factor 1α
tRNA: transfer RNA
