# Supplementary material for: Tissue-Specific Differences in Human Transfer RNA Expression
Source: PLoS Genet. 2006 Dec 22;2(12):e221. doi: 10.1371/journal.pgen.0020221 (PMC1713254; doi:10.1371/journal.pgen.0020221)
Supplement: Supplementary file 1 [file pgen.0020221.sg001.pdf]

Tissue-specific differences in human tRNA expression.

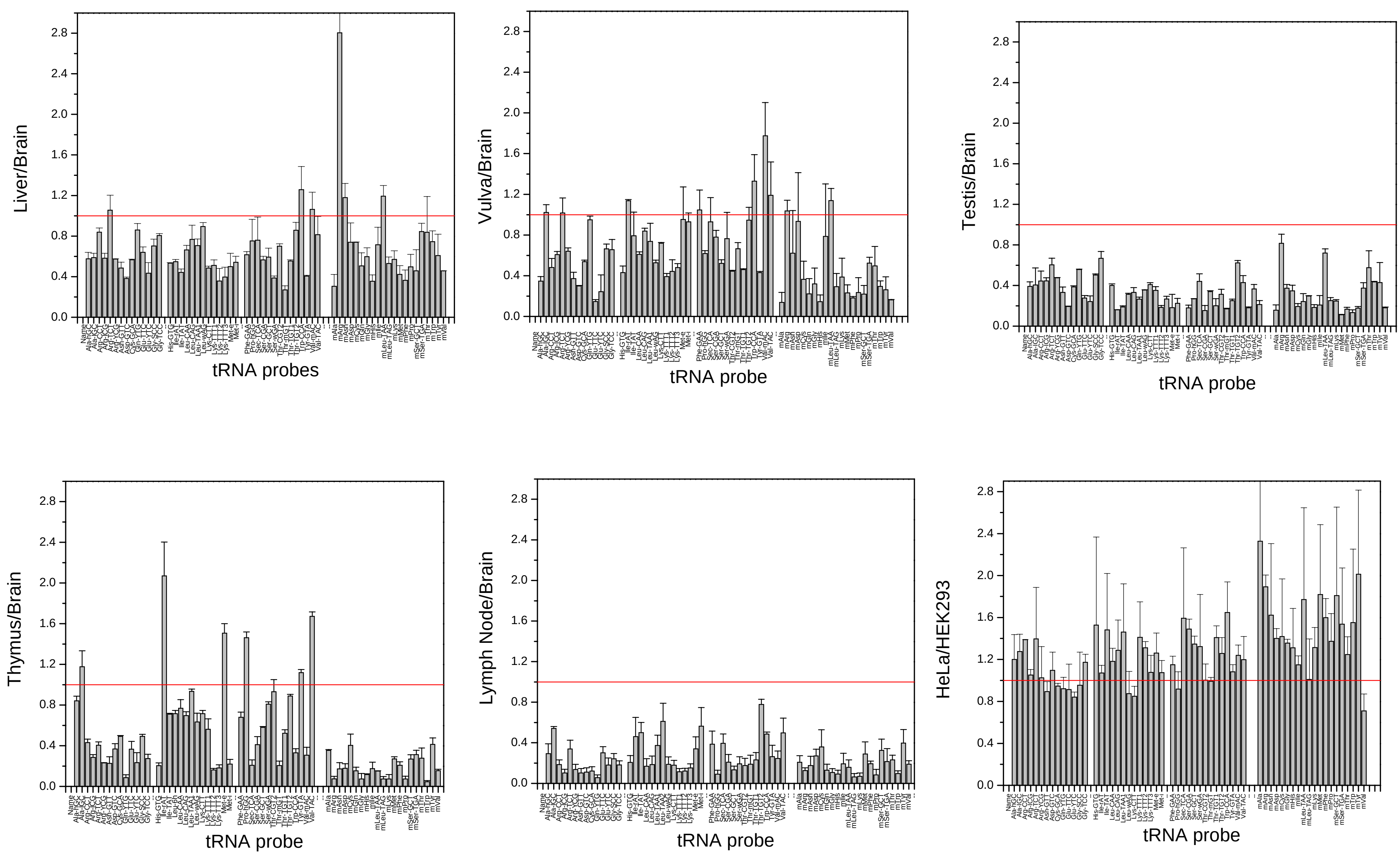

Fig. S1. Relative ratios of each human tRNA probe for liver, vulva, testis, thymus and lymph node versus brain. Bottom right: tRNA abundance in two cell lines.
