# Supplementary material for: Tissue-Specific Differences in Human Transfer RNA Expression
Source: PLoS Genet. 2006 Dec 22;2(12):e221. doi: 10.1371/journal.pgen.0020221 (PMC1713254; doi:10.1371/journal.pgen.0020221)
Supplement: Supplementary file 2 [file pgen.0020221.sg002.pdf]

Tissue-specific differences in human tRNA expression.

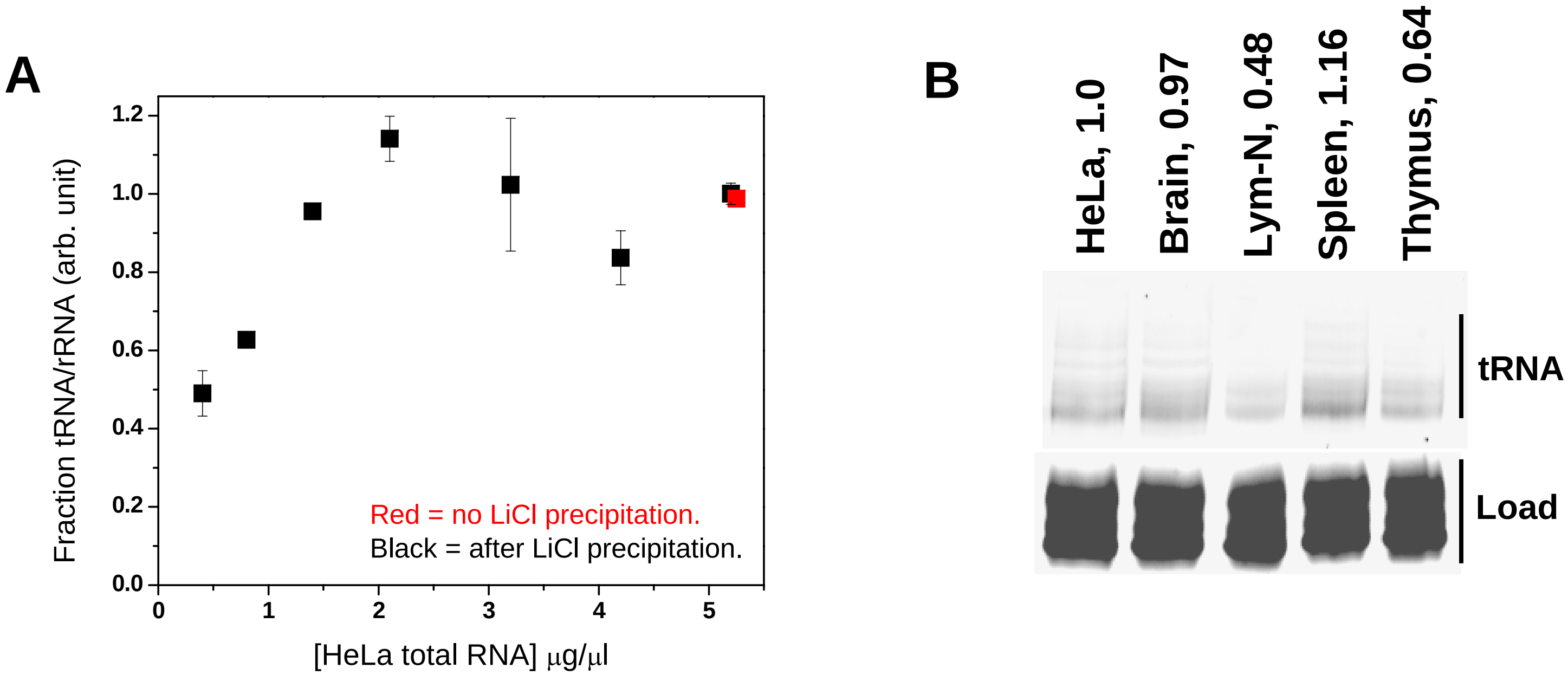

Fig. S2. (A) The ratio of tRNA/(18+28S)rRNA recovered after LiCl precipitation (protocol below) as a function of [RNA] before the addition of LiCl. tRNA and rRNA are separated on 1% agarose gel or 10% denaturing polyacrylamide gels. The ethidium bromide stained gels are scanned using a BioRad phosphorimager and the tRNA and rRNA bands quantified using the BioRad software and averaged. (B) Cy3-labeling of 4 tissue samples and the HeLa total RNA analyzed by denaturing gel. Numbers indicate the amount of labeled RNA relative to HeLa. Consistent with microarray results, the brain and spleen samples produced very similar amount of labeled tRNA as the HeLa sample. The HeLa sample had considerable amount of tRNA as suggested by independent array experiments.

Protocol: The LiCl precipitation was performed using HeLa total RNA isolated using the RNAwiz protocol that does not have this step nor other known steps biasing against tRNAs. The experimental condition was the same as provided by Stratagene: (i) Mix 1 volume of 7.5 M LiCl with 2 volumes of total RNA at high concentration. (ii) Incubate at -20 ºC overnight. (iii) Spin in micro-centrifuge at high speed, remove supernatant. (iv) Wash with ice-cold 70% ethanol. Spin again.
