# Supplementary material for: Tissue-Specific Differences in Human Transfer RNA Expression
Source: PLoS Genet. 2006 Dec 22;2(12):e221. doi: 10.1371/journal.pgen.0020221 (PMC1713254; doi:10.1371/journal.pgen.0020221)
Supplement: Supplementary file 3 [file pgen.0020221.st001.pdf]

Table S1. Human probes for chromosomal encoded tRNA genes.

Probe name (#genes)	Anticodon	Preferred codons
Ala-IGC (21)	IGC	GCU/C
Ala-hGC (21)	IGC(7)+CGC(5)+TGC(9)	GCU/C/A/G
Arg-ICG (7)	ICG	CGU/C
Arg-YCG (11)	CCG(5)+TCG(6)	CGG/A
Arg-CCT (5)	CCT	AGG
Arg-TCT (6)	TCT	AGA
Asn-GTT (24)	GTT	AAU/C
Asp-GTC (15)	GTC	GAU/C
Cys-GCA (29)	GCA	UGU/C
Gln-YTG (20)	CTG(13)+TTG(7)	CAA/G
Glu-YTC (16)	CTC(8)+TTC(4)	GAA/G
Glu-TTC (4)	TTC	GAA
Gly-SCC (19)	CCC(7)+GCC(14)	GGC/U/G
Gly-TCC (9)	TCC	GGA
His-GTG (10)	GTG	CAU/C
Ile-IAT (15)	IAT	AUU/C
Ile-TAT (5)	TAT	AUA
Leu-wAG (12)	IAG(9)+TAG(3)	CUU/C/A
Leu-CAG (9)	CAG	CUG
Leu-CAA (6)	CAA	UUG
Leu-TAA1 (5)	TAA	UUA/G
Leu-TAA2 (1)	TAA	UUA/G
Lys-CTT (12)	CTT	AAG
Lys-TTT1 (12)	TTT	AAA
Lys-TTT2 (1)	TTT	AAA
Lys-TTT3 (1)	TTT	AAA
Met-i (9)	CAT	AUG
Met-e (10)	CAT	AUG
Phe-GAA (11)	GAA	UUU/C
Pro-hGG (21)	IGG(10)+CGG(4)+UGG(7)	CCU/C/A/G
Sec-TCA (1)	TCA	UGA
Ser-CGA (4)	CGA	UCG
Ser-wGA (14)	IGA(10)+TGA(4)	UCU/C/A
Ser-GCT (8)	GCT	AGU/C

Thr-mGT (11)<br>Thr-TGT1 (8)<br>Thr-CGT1 (1)<br>Thr-TGT2 (1)<br>Thr-CGT2 (2)	IGT(9)+CGT(2)<br>TGT<br>CGT<br>TGT<br>CGT	ACU/C/G<br>ACA<br>ACG<br>ACA<br>ACG
Trp-CCA (7)	CCA	UGG
Tyr-GTA (13)	GTA	UAU/C
Val-mAC (25)<br>Val-TAC (5)	IAC(10)+CAC(15)<br>TAC	GUU/C/G<br>GUA

S=C,G; Y=C,T; h=I,C,T; m=I,C;r=I,G; w=I,T.
