# Supplementary material for: Tissue-Specific Differences in Human Transfer RNA Expression
Source: PLoS Genet. 2006 Dec 22;2(12):e221. doi: 10.1371/journal.pgen.0020221 (PMC1713254; doi:10.1371/journal.pgen.0020221)
Supplement: Supplementary file 4 [file pgen.0020221.st002.pdf]

Table S2. Sequences of Human probes for chromosomal encoded tRNA genes.

Probe name	Sequence (5′ – 3′)[a]
Ala-IGC	TGGAGAATG**Y**GGGCGTCGATCCC**R**CTACCTCT**Y**GCATGCTAAGC**R**AGCGCTCTACC**R**CCTGAGCTAATTCCCC
Ala-hGC	TGGAG**R**TGCCGGGGAT**Y**GAACCCGGG**R**CCTC**R**T**R**CATGC**S**AAGCA**Y**GCGCTCTACCACTGAGCTACA**Y**CCCC
Arg-ICG	CGAGCCAGCCAGGAGTCGAACCT**R**GAATCTTCTGATCCGTAGTCAGACGCGTTATCCATTGCGCCACTGGCCC
Arg-YCG	CAACCACG**R**AGGGACTCGAACCCTCAATCTTCTGATCCG**R**A**R**TCAGACGCCTTATCCATTAGGCCACGCGGCC
Arg-CCT	TACCCCAGGTGGGACTCGAACCCACAATCCCTGGCTTAGGAGGCCAGTGCCTTATCCATTAGGCCACTGGGGC
Arg-TCT	CGACTCTGC**Y**GGGACTCGAACCCG**W**AACCTTTGAATTAGAAGTCCAATGCGCTATCCATTGCGCCACAGAGCC
Asn-GTT	CGTCCCTGGGTGGGCTCGAACCACCAACCTTTCGGTTAACAGCCGAACGCGCTAACCGATTGCGCCACAGAGAC
Asp-GTC	CTCCCCGTCGGGGAATCGAACCCCGGTCTCCCGCGTGACAGGCGGGGATACTCACCACTATACTAACGAGGA
Cys-GCA	AGGGGGCACC**Y**GGATTTGAACC**R**GGGACCTCTTGATCTGCAGTCAAATGCTCTACC**M**CTGAGCTATACCCCC
Gln-YTG	AGGTTCCACCGAGA**Y**TTGAACTCGGATCGCTGGATTCA**R**AGTCCAGAGTGCT**H**ACCATTACACCATGG**R**ACC
Glu-YTC	TTCCCTGACCGGGAATCGAACCCGGGCC**WM**GGCGGTGA**R**AGCGCCGAATCCTA**R**CCACTAGACCACCAGGG**R**
Glu-TTC	TTCCCA**Y**ACCGGGAGTCGAACCCGGGCC**R**CCTGGGTGAAAACCAGGAATCCTAACCGCTAGACCAT**R**TGGGA
Gly-SCC	TGCATTGGCC**R**GGAAT**Y**GAACCCGGG**Y**CTCCC**R**CGTGG**W**AGGCGAGAATTCTACCACTG**M**ACCACC**M**A**Y**GC
Gly-TCC	TGCGTTGGCCGGGAATCGAACCCGGGTCAACTGCTTGGAAGGCAGCTATGCTCACCACTATACCACCAACGC
His-GTG	TGCCGTGACTCGGATTCGAACCGAGGTTGCTGCGGCCACAACGCAGAGTACTAACCACTATACGATCACGGC
Ile-IAT	TGGCC**M**GTACGGGGATCGAACCCGCGACCTTGGCGTTATTAGCACCACGCTCTAACCAACTGAGCTAACC**R**GCC
Ile-TAT	TGCTCCAGGTGAGG**M**TCGAACTCACAACCTCGGCATTATAAGTACCGCGCGCTAACCGATTGCGCCACTGGAGC
Leu-wAG	TGGCAG**Y**GGTGGGATTC**R**AACCCACGCC**Y**CCGAA**R**AGACTGGAGCCTTAATCCAGCGCCTTAGACC**R**CTCGGCCAC**R**CTA
Leu-CAG	TGTCA**R**AAGTGGGATTCGAACCCACGCCTCCAG**W**GGAGG**W**TGCGATTTGAACGCAGCGCCTTAAGAC**Y**GCTCGGCCATCC
Leu-CAA	TGTCAGAAGTGGGATTCGAACCCACGCCTCCATTGGAGACCAGAACTTGAGTCTGGCGCCTTAGACCACTCGGCCATCC
Leu-TAA1	TACC**R**GGAGTGGGG**Y**TCGAACCCACGCGGACA**Y**C**S**GTCCATTGGATCTTAAGTCCAACGCCTTAACCACTC**R**GCCATCC
Leu-TAA2	TGTTAA**KR**AGAGGAGTTGAACCTCTGATTATAAA**R**TTTTAAGT**Y**TTATGCAATT**R**CC**R**GGCTCTGCCATCTTAAC
Lys-CTT	CGCCCAACGTGGGGCTCGAACCCACGACCCTGAGATTAAGAGTCTCATGCTCTACCGACTGAGCTAGCCGGGC
Lys-TTT1	CGCC**Y**GAACAGGGACTTGAACCCTGGACCCTCAGATTAAAAGTCTGATGCTCTACC**R**ACTGAGCTATCC**R**GGC
Lys-TTT2	CACCCAAAAAGGGACATGAACCTTGGACCCTCAGATTAAAAGTCTGATGCTCTACTGACTGAGCTATCTGGGT
Lys-TTT3	CGCCTGGACAGGGACTTGAACCCTAGACCCTCAGATTAAAAGTCTGATGTTCTACCTACTGAGCTACCCAGGT
Met-i	TAGCAGAGGATGGTTTCGATCCATCGACCTCTGGGTTATGGGCCCAGCACGCTTCCGCTGCGCCACTCTGCT
Met-e	TGCCC**Y**CTCTGAGGCTCGAACTCAGGACCTTCAGATTATGAGACTGACGCGCTGCC**HR**CTGCGCTAAG**RR**GGC
Phe-GAA	TGC**Y**GAAACCCGGGAT**Y**GAACCAGGGACCTTTAGATCTTCAGTCTAACGCTCTCCCAACTGAGCTATTTCGGC
Pro-hGG	GGGCTCGTCCGGGATTTGAACCCGGGACCTCTCGCACCC**D**AAGCGAGAATCATACCCCTAGACCAACGAGCC
Sec-TCA	TGACCACAAAGGGACTCAAACCCTCAATCTTCTGATCTGAAGTCAGACACCTTATCCATTAAGGCCACACGGTC
Ser-CGA	GCTGTGAGCAGGATTTGAACCTGCGCGGGGA**R**ACCCCATTGGATTTCGAGTCCAACGCCTTAACCACTCGGCCATCACAG
Ser-wGA	GTAG**Y**CGGCAGGATTCGAACCTGCGCGGGGA**R**ACCCCAATGGATTTC**S**AGTCCATCGCCTTAACCACTCGGCCACGACTA
Ser-GCT	GACGAGG**R**TGGGATTCGAACCCACG**Y**GTGCAGAGCACAATGGATTAGCAGTCCATCGCCTTAACCACTCGGCCACCTCGT

Thr-mGT	AGGCCCC**R**CTGGGA**K**TCGAACCCAGGATCTCCTGTTTAC**K**AGACAGGCGCTTTAACCA**R**CTAAGCCA**YR**G**N**GCC
Thr-TGT1	AGGCCCCAGCGAGATT**Y**GAACTCGCGACCCCTGGTTTACAAGACCAGTGCTCTAACC**M**CTGAGCTATGGAGCC
Thr-CGT1	AGGCCCTGGTGAGATTTGAACTCACGACCCCTAGGTTTACGAGACCAGCGCTCCAACCGCTGAGCTACAGGGCC
Thr-TGT2	TGCTGCTCACAGGACTCGAACCTGCATTTCCATCCTTACAAGGGATGTGTAATAGCCAATTATACGAAAGCAGC
Thr-CGT2	AGGCACGGACGGGGTTCGAACCCG**Y**GATCTTCGGTTTACGAGACCGACGCCTTACCACTTGGCCACCGCGCC
Trp-CCA	TGACCCCGACGTGATTTGAACACGCAACCTTCTGATCTGGAGTCAGACGCGCTACCGTTGCGCCACGAGGTC
Tyr-GTA	TCCTTCGAGCCGGA**S**TCGAACCAGCGACCTAAGGATCTACAGTCCTCCGCTCTACCA**R**CTGAGCTATCGAAGG
Val-mAC	TGTTTC**Y**GCC**Y**GGTTTCGAACC**R**GGGACCTTTCGCGTGT**K**AGGCGAACGTGATAACCACTACACTAC**R**GAAAC
Val-TAC	TGGTTCCACTGGGGCTCGAACCCAGGACCTTCTGCGTGTAAAGCAGACGTGATAACCACTACACTATGGAACC
E.coli $tRNA^{Lys}$	TGGGTCGTGCAGGATTCGAACCTGCGACCAATTGATTAAAAGTCAACTGCTCTACCAACTGAGCTAACGACCC
E.coli $tRNA^{Tyr}$	TGGGGGAAGGATTCGAACCTTCGAAGTCGATGACGGCAGATTTACAGTCTGCTCCCTTTGGCCGCTCGGGAACCCCACC
Yeast $tRNA^{Phe}$	TGCGAATTCTGTGGATCGAACACAGGACCTCCAGATCTTCAGTCTGGCGCTCTCCCAACTGAGCTAAATCCGC

a. M = A/C; K = G/T; S = G/C; D = A/G/T; W = A/T; H = A/C/T.
