# Supplementary material for: Tissue-Specific Differences in Human Transfer RNA Expression
Source: PLoS Genet. 2006 Dec 22;2(12):e221. doi: 10.1371/journal.pgen.0020221 (PMC1713254; doi:10.1371/journal.pgen.0020221)
Supplement: Supplementary file 5 [file pgen.0020221.st003.pdf]

Table S3. Human probes for mitochondrial encoded tRNA genes.

probe name	Anticodon	Read codons
mAla-TGC	TGC	GCN
mArg-TCG	TCG	CGN
mAsn-GTT	GTT	AAU/C
mAsp-GTC	GTC	GAU/C
mCys-GCA	GCA	UGU/C
mGln-TTG	TTG	CAA/G
mGlu-TTC	TTC	GAA/G
mGly-TCC	TCC	GGN
mHis-GTG	GTG	CAU/C
mIle-GAT	GAT	AUU/C/A
mLeu-TAG<br>mLeu-TAA	TAG<br>TAA	CUN<br>UUA/G
mLys-TTT	TTT	AAA/G
mMet-CAT	CAT	AUG
mPhe-GAA	GAA	UUU/C
mPro-TGG	TGG	CCN
mSer-TGA<br>mSer-GCT	TGA<br>GCT	UCN<br>AGU/C
mThr-TGT	TGT	ACN
mTrp-TCA	TCA	UGA/G
mTyr-GTA	GTA	UAU/C
mVal-TAC	TAC	GUN
