# Supplementary material for: Tissue-Specific Differences in Human Transfer RNA Expression
Source: PLoS Genet. 2006 Dec 22;2(12):e221. doi: 10.1371/journal.pgen.0020221 (PMC1713254; doi:10.1371/journal.pgen.0020221)
Supplement: Supplementary file 6 [file pgen.0020221.st004.pdf]

Table S4. Human probe sequences for mitochondrial encoded tRNA genes.

**probe name**	**Sequence (5′-3′)**
mAla-TGC	TAAGGACTGCAAAACCCCACTCTGCATCAACTGAACGCAAATCAGCCACTTTAATTAAGCTAAGCCCTT
mArg-TCG	TTGGTAAATATGATTATCATAATTTAATGAGTCGAAATCATTCGTTTTGTTTAAACTATATACCA
mAsn-GTT	CTAGACCAATGGGACTTAAACCCACAAACACTTAGTTAACAGCTAAGCACCCTAATCAACTGG**M**TTCAATCTA
mAsp-GTC	TAAGATATATAGGATTTAGCCTATAATTTAACTTTGACAAAGTTATGAAATGGTTTTTCTAATACCTT
mCys-GCA	AAGCCCCGGCAGGTTTGAAGCTGCTTCTTCGAATTTGCAATTCAATATGAAAATCACCTCGGAGCT
mGln-TTG	CTAGGACTATGAGAATCGAACCCATCCCTGAGAATCCAAAATTCTCCGTGCCACCTATCACACCCCATCCTA
mGly-TCC	TACTCTTTTTTGAATGTTGTCAAAACTAGTTAATTGGAAGTTAACGGTACTATTTATACTAAAAGAGT
mHis-GTG	GGTAAATAAGGGGTCGTAAGCCTCTGTTGTCAGATTCACAATCTGATGTTTTGGTTAAACTATATTTAC
mIle-GAT	TAGAAATAAGGGGGTTTAAGCTCCTATTATTTACTCTATCAAAGTAACTCTTTTATCAGACATATTTCT
mLeu-TAG<br>mLeu-TAA	TACTTTTATTTGGAGTTGCACCAAAATTTTTGGGGCCTAAGACCAATGGATAGCTGTTATCCTTTAAAAGT<br>TGTTAAGAAGAGGAATTGAACCTCTGACTGTAAAGTTTTAAGTTTTATGCGATTACCGGGCTCTGCCATCTTAAC
mLys-TTT	TCACTGTAAAGAGTTGGTTCTCTTAATCTTTAACTTAAAAGGTTAATGCTAAGTTAGCTTTACAGTG
mMet-CAT	TAGTACGGGAAGGGTATAACCAACATTTTCGGGGTATGGGCCCGATAGCTTATTTAGCTGACCTTACT
mPhe-GAA	TGTTTATGGGGTGATGTGAGCCCGTCTAAACATTTTCAGTGTATTGCTTTGAGGAGGTAAGCTACATAAAC
mPro-TGG	TCAGAGAAAAAGTCTTTAACTCCACCATTAGCACCCAAAGCTAAGATTCTAATTTAAACTATTCTCTG
mSer-TGA<br>mSer-GCT	CAAAAAAGGAAGGAATCGAACCCCCCAAAGCTGGTTTCAAGCCAACCCCATGGCCTCCATGACTTTTTC<br>TGAGAAAGCCATGGTAGACATGGGGGCATGAGTTAGCAGTTCTTGTGAGCTTTCTC
mThr-TGT	TGTCCTTGGAAAAAGGTTTTCATCTCCGGTTTACAAGACTGGTGTATTAGTTTATACTACAAGGAC
mTrp-TCA	CAGAAATTAAGTATTGCAACTTACTGAGGGCTTTGAAGGCTCTTGGTCTGTATTTAACCTAAATTTCT
mTyr-GTA	TGGTAAAAAGAGGCCTAACCCCTGTCTTTAGATTTACAGTCCAATGCTTCACTCAGCCATTTTACC
mVal-TAC	TCAGAGCGGTCAAGTTAAGTTGAAATCTCCTAAGTGTAAGTTGGGTGCTTTGTGTTAAGCTACACTCTG
